# Enhancing sustainable development with an intelligent vehicle detection system for a green university^,^

**DOI:** 10.1016/j.mex.2026.104024

**Published:** 2026-06-29

**Authors:** Chartwut Thanajiranthorn, Drusawin Vongpramate, Warawut Chosungnoen, Nuttapol Saenkham

**Affiliations:** Faculty of Science, Buriram Rajabhat University, Thailand

**Keywords:** YOLO, Vehicle counting, Carbon footprint, Sustainable university, SDGs

## Abstract

This study proposes a replicable framework for automated vehicle detection and CO_2_ estimation using YOLOv8 and edge computing. Deployed at Buriram Rajabhat University, the system integrates virtual line-crossing with ByteTrack and Kalman filter stabilization to ensure counting reliability across existing CCTV infrastructure. Performance evaluation showed a 92.3% F1-score, establishing an empirical baseline for campus carbon footprint management and supporting SDGs 11 and 13 through low-cost, AI-driven monitoring.

Integrates YOLOv8 detection with ByteTrack and Kalman filtering for robust real-time vehicle counting on existing surveillance infrastructure.

Incorporates a Cold-start Correction Factor into the emission estimation formula to enhance the accuracy of intra-campus carbon footprint data.

Provides a scalable, low-cost methodological pipeline for academic institutions to monitor sustainability indicators without specialized sensing hardware.

## Graphical abstract

**Figure:**
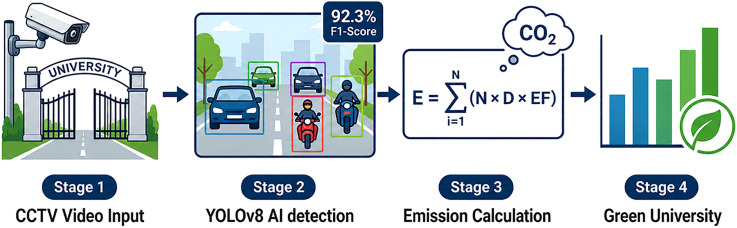

## Specifications table

**Table:** 

**Subject area**	Computer Science
**More specific subject area**	*Environmental Science*
**Name of your method**	*YOLOv8-based Automated Vehicle Counting and* CO_2_*Estimation Framework*
**Name and reference of original method**	*None*
**Resource availability**	*None*

## Background

The global climate emergency is largely driven by increasing greenhouse gas emissions, where the transportation sector is a primary contributor [1,2]. This issue is particularly critical in Thailand, where vehicles significantly impact energy-related emissions [3]. To address this, there is a clear need for effective and scalable monitoring tools that provide reliable data. While many smart city initiatives focus on large urban centers, university campuses like Buriram Rajabhat University (BRU)—which sees over 9000 individuals daily—serve as an ideal environment for developing and validating such tools [4].

This methodology is rooted in the UN's Sustainable Development Goals (SDGs), specifically Goal 11 (Sustainable Cities and Communities) and Goal 13 (Climate Action) [5,6]. The framework integrates existing technologies—YOLOv8, Edge Computing, and established CO₂ emission factors—to create a low-cost, replicable system for academic institutions to track environmental impacts and support "Green University" initiatives.

Unlike conventional traffic monitoring approaches that rely on dedicated traffic sensors or manual counting, the proposed framework integrates computer vision, edge computing, and emission estimation into a unified and deployable monitoring pipeline. The novelty of this method lies in its ability to transform existing campus CCTV infrastructure into an automated environmental monitoring system capable of simultaneously quantifying traffic flow and estimating transportation-related carbon emissions [7]. This integrated workflow provides a scalable and low-cost methodological approach that can be replicated by universities and institutions seeking to monitor sustainability indicators without requiring specialized sensing infrastructure.

## Method details

### Implementation environment and data acquisition

The study utilized the existing CCTV infrastructure at Buriram Rajabhat University, specifically focusing on two main entrance/exit gates. Two IP66-rated cameras were positioned at heights of 4–6 m with a 30–45° downward angle to ensure an optimal field of view for vehicle detection. The video streams (720p resolution) were processed using an Edge Computing unit equipped with an Intel Core i7–8750H CPU, 16 GB RAM, and an NVIDIA GTX 1060 GPU. This decentralized hardware configuration was selected to facilitate real-time, low-latency processing at the data source, thereby minimizing bandwidth requirements for the central network.

On this configuration, the deployed system processed the two 720p camera streams as a single batch on one edge unit. Using a lightweight YOLOv8n detector (640-pixel input, FP16 precision), the complete pipeline (detection, ByteTrack association, Kalman-filter stabilization, and virtual line-crossing logic) sustained 24.7 frames per second per stream, with a mean detection latency of 16.3 ± 1.4 ms for the two-stream batch. During operation the GPU was utilised at 67–74% with a peak memory footprint of 1.8 GB of the 6 GB available, while CPU utilisation remained at 21–26%; the monitoring dashboard refreshed within 85–140 ms and database writes added under 3 ms. These figures confirm real-time operation with substantial headroom on entry-level hardware: the modest GPU load indicates that a single unit could accommodate further streams, and because the YOLOv8 family runs in real time even on low-cost GPUs, the framework can be replicated without high-end accelerators. The principal scalability limitation is GPU throughput per node, so additional gates are accommodated either by adding streams to a node until this budget is reached or by deploying further low-cost nodes, which keeps per-node network bandwidth low.

### System architecture and tracking logic

The core of the detection system utilizes the YOLOv8 architecture [8], the latest model in the YOLO family first introduced by Redmon et al. [9], which provides a high-performance balance between inference speed and detection accuracy. In this implementation, the model was configured with a confidence threshold of 0.2 and a Non-maximum suppression (NMS) mechanism to retain the most confident detections. While the current study utilizes COCO-pretrained weights, future system scalability involves fine-tuning on a localized campus-specific dataset across diverse lighting and weather conditions to elevate detection precision beyond the current 87.5% frame-level detection F1-Score. Recent advancements, such as RT-DETR (Real-Time DEtection TRansformer), offer a promising alternative for complex scenes with high occlusion by leveraging transformer-based architectures that outperform traditional YOLO models in spatial accuracy [10].

For tracking, the system integrates the ByteTrack algorithm and a Kalman Filter to maintain object identities [11,12]. To further minimize ID switching in dense traffic, future iterations may adopt BoT-SORT or StrongSORT, which integrate *Re*-Identification (ReID) features with motion models for more robust associations [1]. Additionally, the framework is designed for the future integration of License Plate Recognition (LPR) using tools like YOLOv10 and Tesseract OCR tailored for Thai scripts, achieving high recognition accuracy in complex environments [13]. This would enable per-vehicle tracking to prevent duplicate counts and allow for more precise emission factors based on specific vehicle data.

### CO_2_ emission estimation

The environmental impact is quantified by converting the automated vehicle counts into CO₂ emission estimates using the following formula:

$$E_{total}=\sum_{i}\left(N_{i}\times D_{i}\times EF_{i}\times CF_{i}\right)$$

Where:

$N_{i}$=Number of vehicles in category $ *i*

$D_{i}$ = Actual travel distance derived from intra-campus survey data (averaging 1.5 km).

$EF_{i}$ = Category-specific emission factors

$CF_{i}$= Cold-start Correction Factor, applied to vehicles exiting the campus (OUT events) to account for the 20–40% higher emission rates during initial engine warm-up [14].

By differentiating between "cold-start" (campus exits) and "hot-stabilized" (entering traffic) states, the methodology provides a more granular environmental impact assessment compared to conventional fixed-factor estimations.

For calibration, the Cold-start Correction Factor was implemented as a single multiplicative coefficient of 1.2 (a 20% increment) applied only to vehicles registering a campus-exit (OUT) event, since such vehicles typically commence their trip from an on-campus parking location and traverse the monitored intra-campus segment while the engine is still in its warm-up phase; entering (IN) vehicles, having reached thermal stabilization on the public road network, were assigned a factor of 1.0. The coefficient was derived from the 20–40% excess-emission range reported for the cold-start phase by Singer et al. [14], adopting the lower bound of this range as a conservative campus-level estimate. Because the magnitude of cold-start excess depends on engine type, ambient temperature, and soak duration, the same coefficient was applied across all vehicle categories as a first-order approximation, and the share of trips treated as cold-start corresponds to the proportion of campus-exit (OUT) events, which accounted for approximately 49.6% of all recorded crossings (3454 of 6966 over the two-week count). Because the gates are located roughly 1.5 km downstream of the campus parking areas, exiting vehicles are already partially warmed by the time they reach the counting line, so the adopted lower-bound factor of 1.2 represents a deliberately conservative estimate of the cold-start contribution. Category-specific and temperature-dependent correction factors, together with a sensitivity analysis of the assumed coefficient (e.g. recomputing total emissions for the 1.0 × lower and upper bounds), are identified as priorities for future refinement.

The emission factors (EF) used in this study were derived from localized research and national standards to ensure regional accuracy:

• Motorcycles: 42.5 g CO₂/km [15].
• Cars (Gasoline): 166.1 g CO₂/km [15].
• Large Vehicles (Buses/Trucks): 512.4 g CO₂/km.

## Method validation

**Fig. 1:**
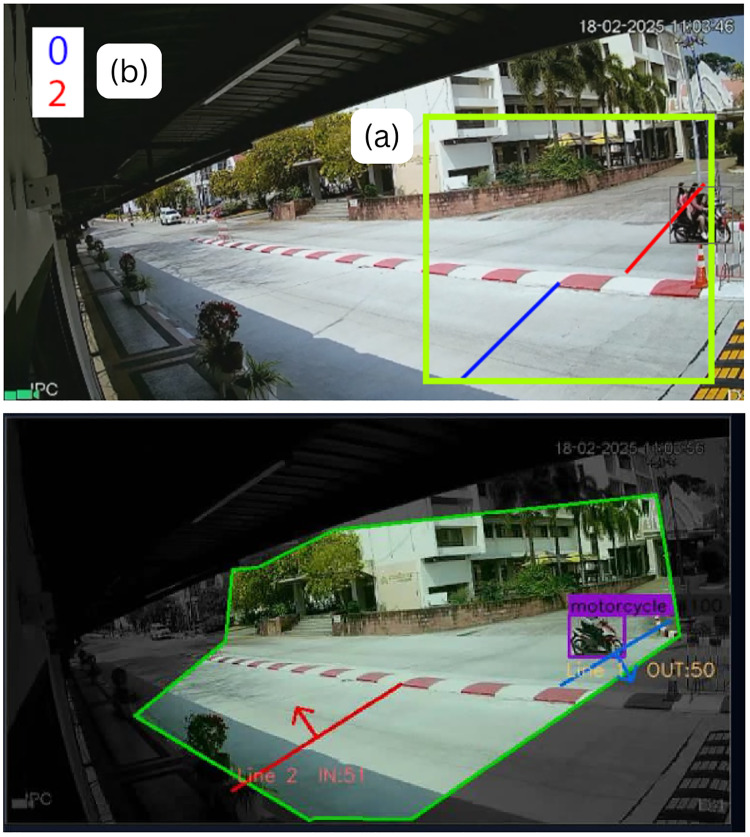
System Interface for Vehicle Counting. (a) Configuration showing the Region of Interest (green box) and virtual lines for entry (blue) and exit (red). (b) Real-time operation, where the system has detected and counted two motorcycles (red counter = 2) exiting the campus. (c) The dual virtual-line counting view, in which Line 2 (red) registers entry events and the second line (blue) registers exit events, with cumulative IN and OUT tallies displayed; a short cool-down delay is applied after each crossing so that a vehicle lingering on a line is not counted more than once.

**Fig. 2:**
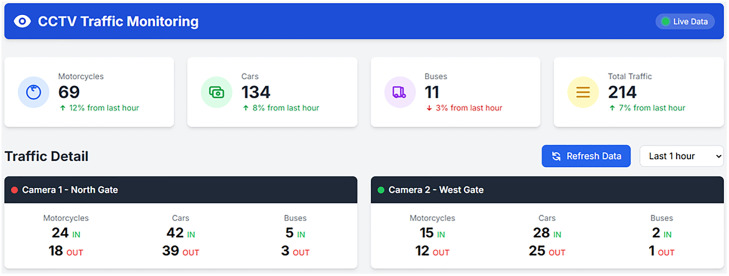
Real-Time CCTV Traffic Monitoring Dashboard. Providing an aggregated view of total traffic and a detailed breakdown of vehicle movements (IN/OUT) for each monitored camera location.

To validate the effectiveness of the proposed monitoring framework, the automated counts were compared against a manually annotated ground-truth dataset. Two annotators independently labelled vehicle crossing events from recorded video, achieving a high inter-annotator agreement (Cohen's kappa = 0.96). This yielded 1135 crossing events spanning five representative operating scenarios (daytime low-volume, morning peak, evening peak with backlight, night-time, and rain) over a total of 240 min, so that performance could be assessed both overall and under individually challenging conditions. Detection and counting performance were evaluated using standard metrics, namely Precision, Recall, and the F1-Score (Fig. 1, Fig. 2).

**Fig. 3:**
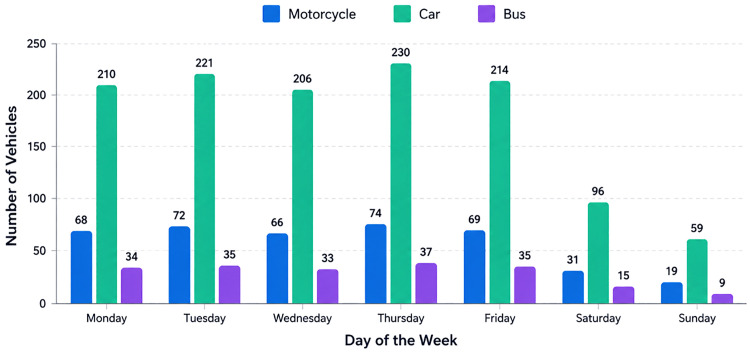
Daily Vehicle Counts by Type. Total number of motorcycles, cars, and buses recorded daily over a one-week period, indicating higher traffic volumes on weekdays.

The system demonstrated high reliability in vehicle flow monitoring, achieving an overall F1-Score of 92.3% across the five validation scenarios. The practical output of this counting accuracy is visualized in Fig. 3, which displays the Daily Vehicle Counts by Type. The data indicates a consistent ability to classify and record motorcycles, cars, and buses over a one-week period, highlighting significantly higher traffic volumes during weekdays compared to weekends.

At the frame level, the YOLOv8n detector achieved an overall F1-Score of 87.5% (Precision 0.887, Recall 0.863), consistent with the use of a lightweight model on 720p footage. Notably, the event-level counting F1-Score (92.3%) exceeded the frame-level detection F1-Score, because in environmental monitoring the temporal consistency of counting is more critical than the spatial precision of any individual bounding box. The integration of the ByteTrack algorithm and Kalman filtering, combined with a 15-frame cool-down that suppresses double-counting, compensated for missed or imprecise frame-level detections by maintaining persistent object identities, thereby preventing significant counting errors [16].

To contextualise these results, the proposed pipeline was compared against alternative vehicle-monitoring approaches. Relative to dedicated inductive-loop or radar sensors—which can achieve comparable counting accuracy but require per-lane installation and recurring maintenance—the present method reuses existing CCTV infrastructure at negligible marginal cost. Relative to manual counting, it removes labour and observer fatigue and enables continuous operation. The contribution of the tracking layer is evident in the gap between detection and counting performance: the frame-level detection F1-Score was 87.5%, whereas the event-level counting F1-Score of the full pipeline rose to 92.3%, confirming that temporal association—rather than frame-level detection precision—is the primary driver of counting reliability. A summary comparison of cost, infrastructure requirements, and reported accuracy against representative published pipelines is provided in Table 1.

**Table 1:** Comparison of the proposed framework with representative vehicle-monitoring and counting approaches.

**Approach**	**Infrastructure & cost**	**Reported accuracy**	**Key limitations**
Proposed framework (this study): CCTV + YOLOv8 + ByteTrack/Kalman on edge units	Reuses existing CCTV; low-cost edge units; no per-lane installation	System-level counting F1 = 92.3%	Frame-level mAP limited by 720p input; needs local retraining for new sites
Single-stage YOLOv8 detection and counting [17]	Camera plus GPU workstation (GTX 1070-class)	Vehicle-counting accuracy 97.5%	Class-level performance varies (e.g. lower recall for buses)
In-pavement inductive-loop detectors [18]	Intrusive in-pavement installation; recurring maintenance; 3–7 yr loop life	Volume error (WMAPE) 4.0–45.5%, depending on install/maintenance	Cannot classify vehicle type; degraded by freeze/thaw and resurfacing
Manual / human-observer counting	No fixed hardware, but high recurring labour cost	Accurate over short periods; degrades with observer fatigue	Not scalable to continuous 24/7 monitoring

Reported accuracy figures are taken from the cited sources and are not directly comparable, as they were obtained on different datasets, hardware, and evaluation protocols; the comparison is indicative of the cost-accuracy trade-off rather than a controlled benchmark.

To assess robustness under challenging conditions, counting performance was analysed separately for each of the five validation scenarios (Table 2). Accuracy was highest under good daytime illumination (S1, F1 = 96.2%) and remained strong during the dense morning peak (S2, 93.5%) and the back-lit evening peak (S3, 91.7%), indicating that the tracking layer largely absorbed the inter-vehicle occlusion that occurs at high traffic density. The largest reductions occurred at night (S4, 86.6%) and during rain (S5, 87.8%), driven mainly by reduced illumination and lower contrast; the night-time subset was also the smallest (67 crossing events), so its estimate carries the widest uncertainty. These results identify night-time and adverse-weather operation as the conditions under which local retraining or higher-resolution capture would yield the greatest benefit.

**Table 2:** Line-crossing counting performance across the five validation scenarios (1135 crossing events; YOLOv8n, 720p).

**Scenario**	**Condition**	**Events**	**Precision**	**Recall**	**F1-Score**
S1	Daytime, low volume (good light)	185	0.957	0.968	0.962
S2	Morning peak (dense traffic)	428	0.929	0.942	0.935
S3	Evening peak (backlight)	312	0.911	0.923	0.917
S4	Night-time (artificial light only)	67	0.866	0.866	0.866
S5	Rain (low light)	143	0.875	0.881	0.878
Overall	All five scenarios	1135	0.918	0.929	0.923

**Fig. 4:**
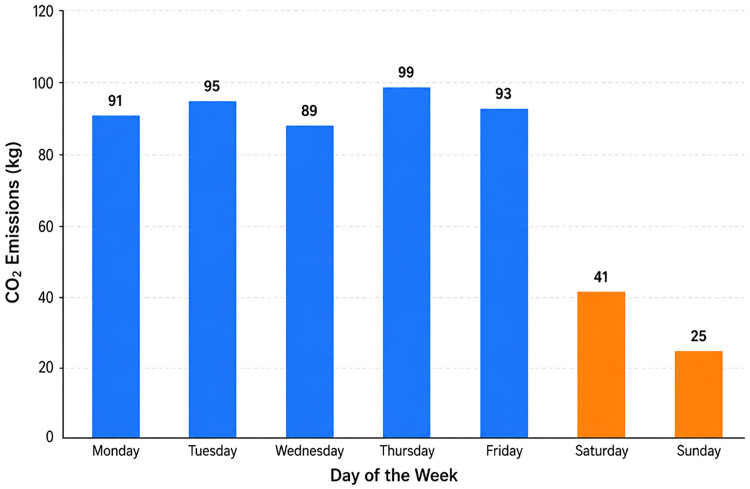
Analysis of Estimated Daily CO₂ Emissions. The calculated daily carbon footprint, visually distinguished between weekdays (blue) and weekends (orange), showing Thursday as the day with the highest emissions and consistently lower emissions over the weekend.

The validated counting data were subsequently used to derive the environmental impact. Fig. 4 presents the Analysis of Estimated Daily CO₂ Emissions, visually distinguishing between weekdays (blue) and weekends (orange). The analysis identifies Thursday as the day with the highest emissions, while showing consistently lower emissions over the weekend. This result confirms that the system can effectively transform raw detection data into actionable sustainability insights. The high system-level accuracy ensures that these CO₂ estimates provide a reliable baseline for university sustainability reporting and data-driven policy making [3].

The system achieved a system-level F1-Score of 92.3%. While the frame-level detection F1-Score was 87.5% due to the 720p resolution of existing CCTV infrastructure, future deployments will transition to **1080p or 4MP cameras**. Higher resolution, combined with **Wide Dynamic Range (WDR)** and top-down viewing angles, significantly reduces detection errors caused by glare and vehicle overlapping (occlusion). The current methodology demonstrates that even with hardware limitations, robust tracking logic can compensate for detection gaps to generate reliable traffic data for institutional sustainability reporting.

## Limitations

Despite the high accuracy of the end-to-end counting system, several limitations were identified during the deployment at Buriram Rajabhat University. While the detector achieved high precision, performance may still be affected by challenging environmental factors such as inconsistent lighting conditions—including shadows or glare—and vehicle occlusion during heavy traffic periods, which can impact the initial detection phase. Furthermore, since the model was primarily tested under local conditions in Buriram, Thailand, its generalizability may be limited; consequently, the system may require retraining with a more diverse, custom campus dataset to maintain accuracy in different geographical locations or extreme weather conditions, such as heavy rain or night-time recording. Regarding the environmental assessment, the CO₂ emission calculation relied on an estimated average travel distance of 1.5 km per trip, an assumption that might not capture the full variation of complex intra-campus mobility patterns. Finally, the scope of the current framework was restricted to CO₂ emissions, excluding other significant vehicle-related pollutants such as Nitrogen Oxides (NO_x_) and Particulate Matter (PM), which represent opportunities for future methodological expansion.

### Generalizability and transferability

Although the framework was validated at a single campus, its design favours transfer to other settings. The methodological pipeline itself—reusing existing CCTV, performing detection with YOLOv8, stabilising counts with ByteTrack and Kalman filtering, and converting validated counts into emissions through a category-wise factor model—is site-independent and can be redeployed wherever camera infrastructure already exists, including other universities, hospital and industrial campuses, and gated urban precincts. Three components, however, are explicitly site-specific and must be re-parameterised rather than reused: (i) the detector, which should be fine-tuned on locally collected imagery to match the prevailing fleet mix, camera geometry, and lighting or weather regime; (ii) the emission factors, which should be replaced with values appropriate to the local vehicle fleet and national reporting standards; and (iii) the average travel distance and cold-start classification, which depend on the spatial layout and parking configuration of the target site. Because these parameters are isolated as explicit inputs rather than embedded in the architecture, adaptation to a new location reduces to a bounded recalibration exercise rather than a redesign. We therefore position the contribution as a transferable, low-cost monitoring template; quantifying performance after transfer to a structurally different site is identified as the natural next step for establishing external validity.

## Ethics statements

The authors confirm that this study was exempted from human research ethics review by the Human Research Ethics Committee, Rajamangala University of Technology Isan (Project Code: HEC-01–68–091) on August 7, 2025. The exemption was granted as the research primarily involved the automated analysis of vehicle traffic through existing CCTV infrastructure and did not involve the collection or processing of personally identifiable information.

## CRediT author statement

**Thanajiranthorn:** Methodology, Writing - original draft. **Saenkham:** Conceptualization. **Vongpramate:** Software. **Chosungnoen:** Writing – review & editing.

## Declaration of generative AI and AI-assisted technologies in the writing process

*During the preparation of this work the authors used Google Gemini in order to refine and improve the English language to meet academic writing standards. After using this tool, the authors reviewed and edited the content as needed and take full responsibility for the content of the published article.*

## Declaration of competing interest

The authors declare that they have no known competing financial interests or personal relationships that could have appeared to influence the work reported in this paper.

## Data Availability

Data will be made available on request.

## References

[1] N. Aharon, R. Orfaig, & B. Bobrovsky (2022). BoT-SORT: robust associations multi-pedestrian tracking. arXiv preprint arXiv:2206.14651.

[2] IPCC (2021).

[3] Technology and Informatics Institute for Sustainable Development (TIIS). (2019). Greenhouse gas emission factors. https://www.nstda-tiis.or.th/lci_and_emission_fac/ghgs-emission-factor/.

[4] Office of Academic Promotion and Registration (2025). https://info.mhesi.go.th/download_stat_s.php.

[5] United Nations. (2015). Goal 11: Sustainable cities and communities. https://sdgs.un.org/goals/goal11.

[6] United Nations. (2015). Goal 13: Climate action. https://sdgs.un.org/goals/goal13.

[7] Majumder M., Wilmot C. (2023). Automated vehicle counting from pre-recorded video using you only look once (YOLO) object detection model. J. Imaging.

[8] G. Jocher, A. Chaurasia, & J. Qiu (2023). Ultralytics YOLOv8 (Version 8.0.0) [Computer software]. https://github.com/ultralytics/ultralytics.

[9] Redmon J., Divvala S., Girshick R., Farhadi A. (2016). Proceedings of the IEEE Conference on Computer Vision and Pattern Recognition (CVPR).

[10] Y. Zhao, W. Lv, S. Xu, J. Wei, G. Wang, Q. Dang, Y. Yi, J. Chen, & H. Cheng (2024). DETRs Beat YOLOs on real-time object detection (RT-DETR). arXiv preprint arXiv:2304.08069.

[11] Kalman R.E. (1960). A new approach to linear filtering and prediction problems. J. Basic Eng..

[12] Zhang Y., Sun P., Jiang Y., Yu D., Weng F., Yuan Z., Luo P., Liu W., Wang X. (2022). Proceedings of the European Conference on Computer Vision (ECCV).

[13] Meesad P., Thumthong W. (2025). Advanced deep learning techniques for automated license plate recognition. Sci. Rep..

[14] Singer B.C., Kirchstetter T.W., Harley R.A., Kendall G.R., Hesson J.M. (1999). A fuel-based approach to estimating motor vehicle cold-start emissions. J. Air Waste Manag. Assoc..

[15] Nilrit S., Sampanpanish P. (2012). Emission factor of carbon dioxide from In-use vehicles in Thailand. Mod Appl Sci.

[16] Ren P., Wei F., Djahel S. (2017). *2017 IEEE* International *Smart Cities Conference (ISC2)*.

[17] Neamah S., Karim A. (2024). Vehicle classification and counting for traffic analysis based on single-stage YOLOv8 model. Iraqi J. Comput. Commun. Control Syst. Eng..

[18] National Academies of Sciences, Engineering, and Medicine (2025).

